# Evaluation of different doses of medetomidine for the induction of emesis in cats

**DOI:** 10.1177/1098612X251367617

**Published:** 2025-09-28

**Authors:** Florian Sänger, René Dörfelt

**Affiliations:** Ludwig-Maximilians-Universität München Small Animal Clinic, Centre for Clinical Veterinary Medicine, Faculty of Veterinary Medicine, Ludwig-Maximilians-Universität München, Munich, Germany

**Keywords:** Intoxication, decontamination, foreign body ingestion, vomitus, alpha_2_-agonist

## Abstract

**Objectives:**

This study aimed to investigate the emetic effect of medetomidine, determine the best dose for clinical practice and investigate any adverse effects at different doses.

**Methods:**

In this prospective, observational study, 10, 20, 30 and 40 µg/kg of medetomidine was administered intramuscularly (IM) to cats after ingestion of foreign substances, to induce emesis. The success rate, frequency, time after injection, sedation score and adverse effects were recorded. If induction of emesis was not successful after 10 or 20 µg/kg medetomidine, a second bolus of medetomidine (20 µg/kg IM) was administered 10 mins later.

**Results:**

A total of 58 cats were included after foreign material ingestion. Emesis was achieved with all doses of medetomidine (39/58), whereby the highest success rate was reached with 20 µg/kg (13/17). The success rate after the first injection was not statistically different between the different medetomidine doses (*P* = 0.457). The median time to emesis after the first injection of medetomidine for all cats was 5 mins (range 3–14). The sedation score was lower after 10 µg/kg medetomidine compared with 40 µg/kg (*P* = 0.013). The most common adverse effect was sedation. Medetomidine was antagonised with atipamezole in 47/58 cats.

**Conclusions and relevance:**

Medetomidine is an effective drug for inducing emesis in cats and is a reasonable alternative to other alpha_2_-agonists. The most successful dose with the least adverse effects was 20 µg/kg IM.

## Introduction

The observed or suspected ingestion of poisonous substances or foreign material is an uncommon but potentially life-threatening cause of presentation of cats to the veterinary practice. In a retrospective study, 166 cats with suspected poisoning were presented to a referral centre over 5 years.^1^ After an initial examination and stabilisation, the aim of treatment is often to remove the foreign material from the stomach to prevent further absorption of the toxicant or to prevent the foreign body from being transported further into the intestinal tract and causing an obstruction.^2^ A standard method of decontamination is the induction of emesis. In dogs, this is typically achieved with apomorphine or ropinirole.^3–6^ Various drugs are used for inducing emesis in cats, such as alpha_2_ (α_2_)-agonists or hydromorphone, with the α_2_-agonists xylazine and dexmedetomidine being the most common.^2,7–9^ In addition to injections, oral administration of dexmedetomidine has previously been evaluated.^10^ Another α_2_-agonist frequently used in veterinary practice is medetomidine. Medetomidine is licensed for sedation in doses up to 80 µg/kg IM.^11^ In studies evaluating the emetic effect of xylazine, the standard emetic drug for cats, success rates of 43–60% were achieved with median doses of xylazine of 0.36–0.64 mg/kg IM.^2,7,12^ Dexmedetomidine, the dextrorotatory optical isomer of medetomidine, causes nearly all of the pharmacological effects of medetomidine.^13^ The equipotent dose of dexmedetomidine for sedation, analgesia and muscular relaxation is approximately 50% of the medetomidine dose.^13^ Only at very high doses of 50–75 µg/kg does dexmedetomidine have a slightly longer sedative effect compared with the equipotent dose of medetomidine, probably due to a greater α_1_-adrenoceptor agonistic effect of medetomidine.^13^ Retrospective studies evaluating the emetic effect of dexmedetomidine in cats had success rates in the range of 58–81% using doses of 6–18 µg/kg.^2,8,12^

Medetomidine is also empirically used to induce emesis in cats, but its emetic effect has not been studied so far.^9^ Furthermore, no scientific data exist on the most effective emetic dose of medetomidine.

This study aimed to investigate the emetic effect of medetomidine in cats after ingestion of foreign material, and to determine the dose that produced the strongest emetic effect with minimal adverse effects. We hypothesised that medetomidine would be suitable for inducing emesis in cats, and that higher doses would yield a greater emetic success rate compared with lower doses.

## Materials and methods

### Ethical approval

This study was approved by the ethical committee of the Center of Clinical Veterinary Medicine of the Ludwig-Maximilians-University Munich (number 280-21-07-2021). Owners signed an informed consent form before participating in the study.

### Inclusion criteria

The study population consisted of client-owned cats presented after foreign material ingestion. Cats were included if the clinician decided to aim for removal of the material by inducing emesis.

### Exclusion criteria

Cats were excluded if their mental status was already altered due to signs of poisoning or if they had ingested material for which emesis is contraindicated (eg, petroleum, detergents, corrosive substances or sharp objects, potentially injuring the gastrointestinal tract during emesis). A history or clinical signs of severe underlying diseases, such as chronic kidney disease or decompensated cardiac diseases, also led to exclusion from the study.

### Study design

**Figure 1:**
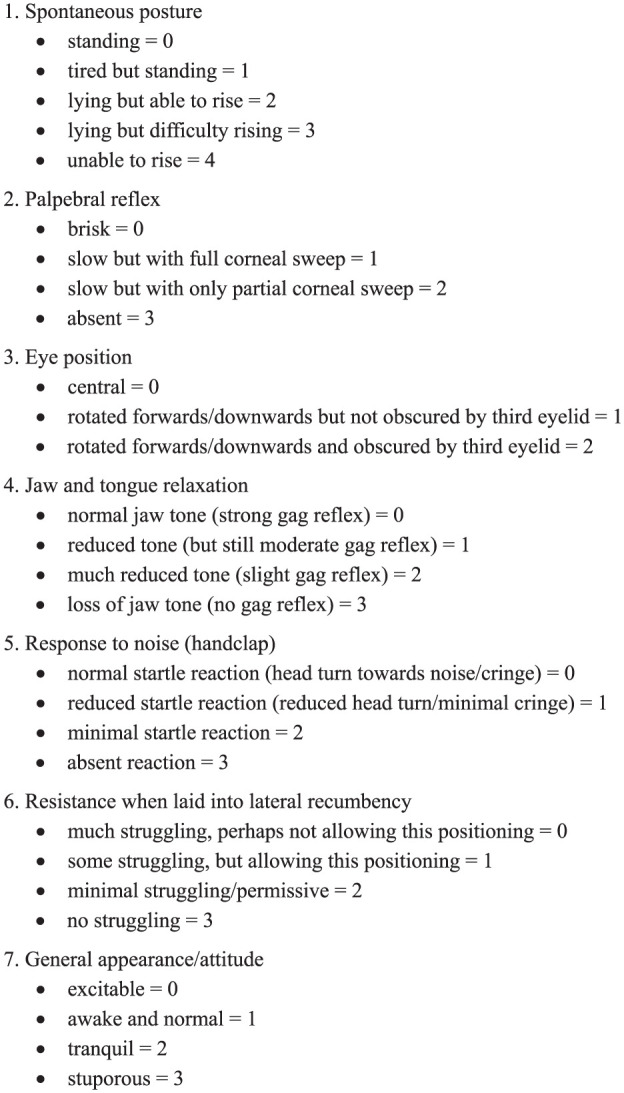
Sedation scale (from Grint et al^14^)

History-taking and clinical examination were performed in all cats included in this prospective, observational clinical study. Emesis was then induced with medetomidine (Sedator; Dechra Pharmaceuticals). Cats were assigned to one of four dose groups by quasi-randomisation with alternating allocation: group 10 received medetomidine 10 µg/kg IM; group 20 received 20 µg/kg IM; group 30 received 30 µg/kg IM; and group 40 received 40 µg/kg IM. After the first 40 cats, the study population was extended, but no further randomisation was performed. The time from ingestion of foreign material to presentation, success rate, frequency and time to emesis, sedation scale score and adverse effects were recorded for all groups. If induction of emesis was not successful in group 10 or group 20 after 10 mins, a second bolus of medetomidine 20 µg/kg IM was administered and the success rate, frequency and time to emesis after the first injection was evaluated for the cumulative dose. At 20 mins after the last bolus of medetomidine, a second clinical examination was performed. The sedation scale score (Figure 1) was calculated as previously described by Grint et al^14^ at this time point. If cats vomited earlier, a clinical examination was performed 5 mins after they stopped vomiting. If patients were moderately or severely sedated, the effect of medetomidine was antagonised with atipamezole (Revertor; CP-Pharma). Atipamezole was administered after the second clinical examination, if necessary.

### Statistical analysis

Statistical analysis was performed using commercially available software (Prism 5 for Windows; GraphPad Software). Data were evaluated for normality with the D’Agostino and Pearson normality test. Parametric data were reported as mean ± SD. Non-parametric data were reported as median and range. Differences in the success of emesis between groups were analysed using Fisher’s exact test. Differences in time since ingestion, time to emesis, frequency of emesis and sedation scale score between groups were analysed using the Kruskal–Wallis test with post-hoc Dunn’s multiple comparison test. A *P* value ⩽0.05 was considered significant.

## Results

The study population consisted of 58 cats with foreign material ingestion. Of them, three were intact males, 32 were neutered males, seven were intact females and 16 were spayed females. The breed distribution of the cats consisted of domestic shorthair (33/58), British Shorthair (14/58), Maine Coon (2/58), Scottish Fold (2/58), Ragdoll (2/58), Turkish Angora (2/58) and one of each of the breeds Russian Blue, Neva Masquerade and Siberian Forest cat. The median age was 2.0 years (range 0.25–16.0) and the mean weight was 4.4 ± 1.3 kg.

Cats were presented with ingestion of plants, mostly lilies (16/58; lilies 8/16), linear foreign bodies (15/58), inappropriate human food such as chocolate, raisins or grapes (12/58), human drugs (3/58) and other foreign material (12/58). The median time from ingestion of foreign material to presentation was 60 mins (range 7–900).

**Figure 2:**
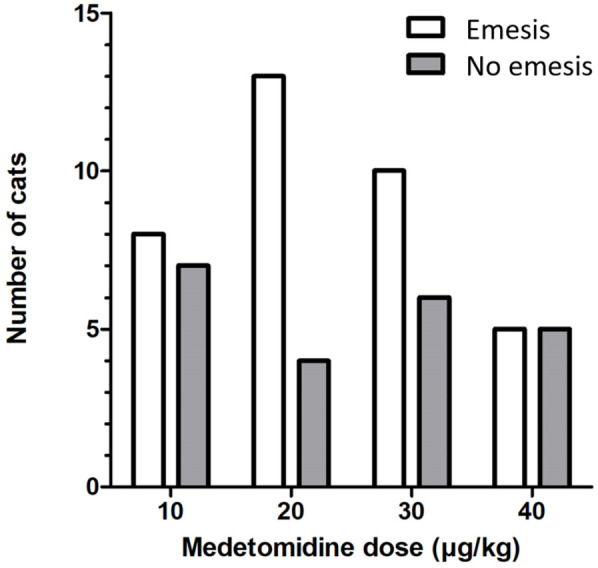
Success rate for inducing emesis in 58 cats after the first injection of medetomidine in the different dose groups

The overall success rate for emesis induction after the first injection was 62.1% (36/58 cats). A second dose of medetomidine was required in seven cats in group 10 and four cats in group 20. Of these cats, three vomited after the second dose. The success of inducing emesis after the first and second doses of medetomidine, and therefore after the cumulative medetomidine dose, was not statistically different between groups (*P* = 0.457, *P* = 0.158) (Table 1, Figure 2).

**Table 1:** Comparison of different doses of intramuscular medetomidine for induction of emesis in 58 cats

	Medetomidine 10 µg/kg		Medetomidine 20 µg/kg		Medetomidine 30 µg/kg		Medetomidine 40 µg/kg		*P* value
	N	Value	N	Value	N	Value	N	Value	
Success rate after first injection (%)	15	8 (53.3)	17	13 (76.5)	16	10 (62.5)	10	5 (50.0)	0.457
Success rate with cumulative dose (%)	15	9 (60.0)	17	15 (88.2)	–	–	–	–	0.158
Frequency of emesis after first injection	15	0 (0–3)	17	2 (0–5)	16	1 (0–4)	10	0.5 (0–2)	0.050
Frequency of emesis after cumulative dose	15	1 (0–3)	17	2 (0–5)	–	–	–	–	0.106
Time to emesis after injection of medetomidine (mins)	15	7.5 (2–10)	17	5 (3–7)	16	6 (2–14)	10	3 (3–5)	**0.041**
Time to emesis after injection of cumulative dose of medetomidine (mins)	15	8 (2–13)	17	5 (3–15)	–	–	–	–	0.251
Sedation scale score	15	7 (1–17)	17	10 (1–19)	16	10 (2–17)	10	16 (10–19)	0.013^*^

Data are presented as median and range. Statistically significant values are printed in bold

* Significantly different between 10 and 40 µg/kg

N = number of patients

The median frequency of emesis after the first injection for all cats was 1.0 (range 0–5.0) and was significantly different between groups (*P* = 0.05). The frequency of emesis with the cumulative medetomidine dose was not significantly different between the groups (Table 1).

The median time to emesis after the first injection of medetomidine for all cats was 5 mins (range 3–14). The median time after the first injection of medetomidine was 7.5 mins (range 2–10) in group 10 and 3 mins (range 3–5) in group 40, and was significantly different between the groups (*P* = 0.041). The median time to emesis after injection with the cumulative dose was 5 mins (range 3–20). The median time after injection with the cumulative dose was not significantly different between the groups (Table 1).

**Figure 3:**
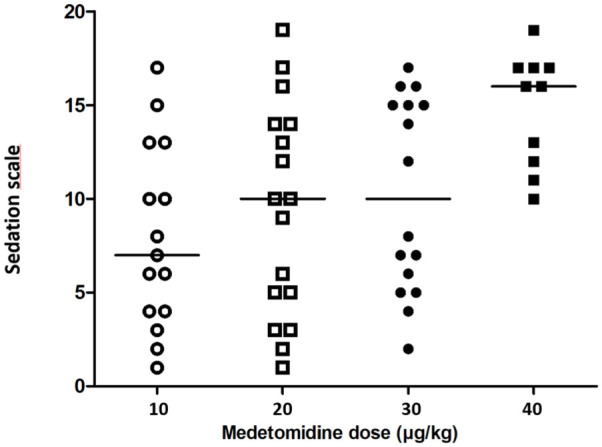
Comparison of sedation score in 58 cats after administration of medetomidine for emesis induction at two different doses of medetomidine

The median sedation score for all cats was 12 (range 1–19). When comparing between groups, the sedation score was significantly lower in group 10 compared with group 40 (*P* = 0.013) (Figure 3, Table 1).

Besides sedation, 4/58 (6.9%) cats developed bradycardia with a heart rate <120 beats/min. Bradycardia lasted for a few minutes and none of the cats required an intervention besides antagonisation with atipamezole. No other adverse effects were recorded. Medetomidine was antagonised with atipamezole in 47/58 (81.0%) cats.

## Discussion

In the present study, the emetic effect of medetomidine and its adverse effects were evaluated. The results indicate that medetomidine is an effective medication for the induction of emesis in cats and it was successful with a dose of 10–40 µg/kg IM in 62.1% of the cats. Success rates did not increase with higher doses of medetomidine.

The most common drugs used for induction of emesis in cats are α_2_-agonists.^2,7,12^ The emetic effect of α_2_-agonists is generated by stimulating α_2_-adrenoceptors in the chemoreceptor trigger zone of the area postrema.^7^ Medetomidine acts on α_2_-adrenoceptors, in a similar way to xylazine, but medetomidine has a higher selectivity for α_2_-adrenoceptors compared with xylazine.^15^ Therefore, medetomidine is a viable alternative to the use of xylazine for induction of emesis in cats. In addition, medetomidine is more likely to be readily available than xylazine in small animal practices, as xylazine is licensed only for large animals in some countries.

The highest success rate for induction of emesis (76.5%) was achieved with 20 µg/kg. With a second injection of 20 µg/kg medetomidine 10 mins later for non-vomiting cats, the success rate increased to 88.2%. The success rates in the other groups were lower, at 50.0%, 53.3% and 62.5%, respectively. In the 40 µg/kg group, the sedative effect occurred within a few minutes, which might explain the lower success rate in this group. Even though more cats vomited in the 20 µg/kg group, no statistically significant difference in the success of induced emesis was found between the groups, most likely due to the low number of patients. For xylazine, a dose-dependent effect on the induction of emesis could be observed, with an effective dose (ED_50_) of 0.277 mg/kg IM and an ED_100_ of 1 mg/kg IM.^16^ This dose-dependent effect could not be observed for medetomidine in this study. The reason for this might also be the faster sedative effect in the 30 and 40 µg/kg groups.

If the first bolus did not successfully induce emesis in groups 10 and 20, a second bolus of medetomidine was applied. This cumulative dose did lead to higher success rates of 60.0% and 88.2% in groups 10 and 20, respectively. Therefore, it might be a reasonable option to give a second bolus of medetomidine to a clinical patient 10 mins after the first bolus if the first bolus did not induce emesis.

The most frequently observed adverse effect in this study was sedation. The sedation score increased with increasing doses of medetomidine, with the highest sedation score in group 40. The only other adverse effect was bradycardia. In another study evaluating the emetic effect of xylazine and dexmedetomidine, 2/25 cats receiving xylazine and 1/16 cats receiving dexmedetomidine were sedated. No other adverse effects were recorded.^2^ In the study by Thies et al,^7^ 16/48 (33%) cats had adverse effects, with sedation being the most frequently observed adverse effect (15/48) in cats receiving xylazine. In the study by Willey et al,^12^ the sedation level was not recorded consistently and was classified as moderate to excessive. A certain degree of sedation must be tolerated to achieve a sufficient emetic effect. As the sedative effect can be antagonised with atipamezole, its management in clinical practice is feasible.

The present study has some limitations. First, the number of cats per group is small. This might explain the lack of a statistically significant difference in success rates between the groups. Other retrospective studies evaluating the emetic effect of α_2_-agonists had similar patient numbers, with 43, 47 and 48 cats, respectively.^2,7,12^ However, these studies did not compare different doses.

A cumulative dose with a second bolus of medetomidine was evaluated only in groups 10 and 20. In these groups, a higher success rate of emesis could be achieved with the second bolus. There might also have been a higher success rate of emesis in groups 30 and 40 with a second bolus of medetomidine. This was not applied in these groups, as these cats had already received high doses, with a higher potential of sedation at the application of the second dose, potentially associated with a higher risk of aspiration. In groups 10 and 20, only 3/11 cats that received a second bolus of medetomidine vomited. Therefore, it is questionable whether a second bolus in groups 30 and 40 would have resulted in a significantly higher success rate. It is more likely that the already present sedation would have worsened.

The median time from ingestion of foreign material to presentation was 60 mins, but the study population also included cats with a longer duration since ingestion (up to 900 mins). It cannot be excluded that in some cats, the foreign material was no longer in the stomach at the time of presentation. An already empty stomach might have led to some unsuccessful attempts to induce emesis in these cats with a longer time since ingestion.

Cats were monitored for only 20 mins after the last bolus of medetomidine or for 5 mins after successful emesis, and were treated with atipamezole if they were severely sedated. After antagonisation, or in the absence of adverse effects, they were discharged. It cannot be excluded that any other adverse effects occurred after discharge.

## Conclusions

Medetomidine is an effective drug for inducing emesis in cats. The dose of 20 µg/kg IM in particular seems to have the highest emetic effect with a comparably low sedative effect. Medetomidine has a comparable success rate for inducing emesis in cats to that reported in other studies for xylazine and dexmedetomidine, and is therefore a feasible drug for clinical use.
